# To Stage or Not to Stage? Direct-to-Implant Versus Staged Reconstruction for Implant-Based Breast Reconstruction

**DOI:** 10.1093/asjof/ojae007.080

**Published:** 2024-04-12

**Authors:** Charalampos Siotos, Kelly Harmon, Nikki Rezania, Deana Shenaq

**Affiliations:** Rush University Medical Center, Chicago, IL; Rush University Medical Center, Chicago, IL; Rush University Medical Center, Chicago, IL; Rush University Medical Center, Chicago, IL

## Abstract

**Goals/Purpose:**

Implant-based breast reconstruction remains the common method of reconstruction after mastectomy in the US. An increasing number of centers are now offering direct-to-implant (DTI) breast reconstruction, avoiding staged reconstructions with tissue expander placement first. However, little is known regarding the need for revisions following DTI. In this study we sought to investigate short- and long-term complications and revisions following DTI versus staged reconstruction.

**Methods/Technique:**

We retrospectively reviewed all patients who underwent bilateral nipple-sparing-mastectomies seeking implant-based breast reconstruction between September 2016 to September 2021. We extracted demographic and clinical information for the selected patients. Patients were divided in two cohorts: the DTI cohort, and the staged cohort. Baseline differences among the two groups were assessed by performing non-parametric statistical tests. Number of complications and revisions were assessed by employing chi-square. Logistic regression was then employed to adjust for possible confounders.

**Results/Complications:**

During the study period, we identified 143 patients, 69 in the DTI cohort and 74 in the staged cohort. Patients in the staged cohort were more likely to have higher BMI (mean 26 kg/m^2^ versus 24.3 kg/m^2^ in the DTI cohort) and have undergone prior radiotherapy than those in the DTI cohort. No differences based on age, race/ethnicity or comorbidities were present. According to our analysis and within the follow up period time of average 21 months, the two cohorts had similar rates of minor and major short- and long-term surgical complications. After adjusting for potential confounding factors, the DTI group had a significantly higher rate of overall revisions (OR 2.73, 95% CI 1.23-6.07, p-value 0.01) and specifically revisions with implant exchange (0R 3.06, 95% CI 1.06-8.85, p-value 0.03). Most common reason for reason was asymmetry or contour deformity. However, the DTI cohort had significantly lower charges associated with their care during the follow up period (d=-100,178.14 US dollars, p-value=0.001). Complications rates were similar among the two groups.

**Conclusion:**

An increasing number of patients are now interested in DTI breast reconstruction. According to the results of our study, DTI patients may have increased number of revisions but share similar rates of complications. DTI breast reconstruction is also associated with lower charges, even after accounting for the additional necessary revision procedures. Careful selection of patients for DTI and appropriate education on the need for revisions in the future is paramount.

